# The nutritional content of Tana River yellow baboon (*Papio cynocephalus*) foods in a partially forested habitat

**DOI:** 10.1371/journal.pone.0207186

**Published:** 2018-11-15

**Authors:** Vicki K. Bentley-Condit, Michael L. Power

**Affiliations:** 1 Department of Anthropology, Grinnell College, Grinnell, Iowa, United States of America; 2 Nutrition Laboratory, Smithsonian Conservation Biology Institute, National Zoological Park, Washington, D.C., United States of America; Universidad Nacional Autonoma de Mexico Instituto de Investigaciones en Ecosistemas y Sustentabilidad, MEXICO

## Abstract

Here we report the first dietary macronutrient and mineral content information for a little-studied yellow baboon group (i.e., the Mchelelo troop) at the Tana River Primate National Reserve, Kenya. We compare forest to savanna samples for this troop found in a partially forested habitat. Observations conducted between 1988 and 1992 determined our list of foods. Subsequently, flora samples, representing 56 species, were collected between April 2008 and March 2009 with nutrient content determined via standard procedures for fiber, gross energy, ash/minerals, crude protein, and crude fat/lipids. Concentrations of specific minerals (calcium, iron, magnesium, manganese, phosphorus, potassium, zinc) were also measured. We predicted forest items would be higher in gross energy and lipids and savanna items higher in crude protein, fiber, and minerals. Our analyses support only the predicted difference in crude protein for savanna items for the overall dataset. In our examination of the top 15 foods, savanna items had significantly higher crude protein, ash, magnesium, and manganese while forest items had higher gross energy. Right-angled mixture triangles show some clustering by location but with substantial overlap in values. Our data provide further indication of the particularity and purposefulness of dietary choices made by primates. They also contribute to the broader discussions of primate nutritional ecology and are a first step towards an examination of macronutrient balancing for this group. Finally, we discuss the impact heavy reliance upon forest products by a “savanna species” may have upon competitors and forest composition. Ultimately, we show that there is still much to be learned about baboon nutrition.

## Introduction

The nutritional content of food items is an important aspect of primate behavioral ecology. The National Research Council guidelines [1], compiled from numerous studies of primate feeding ecology, list 240 primate species or subspecies–either captive or wild–that were the subjects of feeding ecology research conducted prior to, or during, 1998 (see also [2–8]). Despite this seeming abundance of research, there are still many gaps in our knowledge of the nutritional content of food items consumed by wild populations.

Baboons are known to be eclectic yet selective feeders–consuming a wide variety of food items in their efforts to meet their daily needs. Over roughly the past five decades, researchers have published dietary data for at least 18 baboon groups beginning with Rowell’s [9] early olive baboon work in Queen Elizabeth National Park (see also [10–13] and Hill and Dunbar’s [14] table summarizing the broad dietary choice results of most of these studies). However, to date, data regarding nutritional content of foods consumed by only a handful of these populations (Amboseli, Cape Peninsula, Drakensberg, Laikipia, Mikumi, and Kuiseb/Okavango) have been published [10–12, 15–17].

Where these nutritional data are available, they have generally shown baboon diets to exhibit broad trends similar to patterns seen in other primate species; baboons tend to select foods higher in proteins and lipids and lower in fiber, tannin, and alkaloid content [12]. Even so, we cannot and should not assume that all baboon diets are the same or make general assumptions regarding savanna baboons’ food choices (e.g., fruit being higher quality and leaves being lower quality options) [17] as there is much variation between habitats, species, and groups. Thus, it is still important to investigate baboon nutrition, particularly for little-studied groups.

The baboon research cited in the previous paragraphs [9–17] provides data on nutrient intake and balancing by the baboons studied in addition to nutrient content. While we are unable to address nutrient intake (see Materials and Methods), here, we provide nutrient content data for select foods consumed by the Mchelelo troop of yellow baboons (*Papio cynocephalus*) at the Tana River Primate National Reserve (TRNPR), Kenya. The only other nutrition data reported for this group to date discuss food consumption alone [18–19]. Our nutrient content data for this group of wild primates are an integral component of the broader topic of primate nutritional ecology. Understanding a group’s nutrition choices can, in turn, contribute to a broader understanding of behavior. For example, Coogan & Raubenheimer [20] use grizzly bear macronutrient balancing data to explore grizzly bear-human conflict. A similar approach could be useful in exploring nonhuman primate-human conflicts or in investigating the impact of one primate species upon another sympatric species. The point here is that there are likely nutritional underpinnings to primate behaviors including conflicts and habitat usage.

For example, research is emerging that highlights the utilization of forest environments by both “savanna” baboons [21–22] and those classified as “forest-living” [23]. These studies emphasize the mosaic environment inhabited and exploited by baboons and the selectivity they show in meeting their nutritional needs within those environments.

One point to emerge from Bentley-Condit’s [19] preliminary inventory of flora consumed by the Mchelelo baboon troop was that approximately 42% of the food consumption observations occurred in the forest. As the 63ha forest area used regularly by this troop represented only 8.7% of their entire regularly used area, their consumption of forest products was considerable and their use of forest habitat was non-random (Ivlev’s electivity index = 0.66 [24–25]). This level of forest habitat preference by yellow baboons has not been reported elsewhere, although Kunz and Linsenmair [22], Nagel [21], and Rowell [9] each reported similar non-random usage of forest habitats by olive baboon groups in the Ivory Coast, Ethiopia, and Uganda respectively. In fact, Kunz and Linsenmair [26] found that the olive baboons at the Comoé National Park in the Ivory Coast were quite frugivorous, with almost half of their feeding time devoted to fruits and seeds. Because fruits tend to be more abundant in forests than on the savanna [27–30], it is not surprising that baboons with access to forests may include more fruit species. It was our finding of non-random forest usage by the Mchelelo baboons, coupled with an overall lack of nutritional data for this population, that led us to conduct this research to examine the importance of the forests and forest foods to Mchelelo baboon diet.

Thus, we focus our examination of nutrient content on a comparison of forest to savanna food items. We do not have complete dietary data for the Mchelelo baboons (see Materials and Methods below for details). However, for the samples we possess, we propose that forest and savanna food items’ nutritional make-up should differ, in both macronutrients and minerals. We predict the forest items, collectively, will be higher in gross energy (GE) and lipids (due to a higher percentage of fruits/seeds) while the savanna items, again collectively, will be characterized by higher protein and fiber (due to a higher percentage of grasses). These predictions are based on the proportions of items from fruit/seeds vs. grasses found in these respective locations, not on any particular item that might be found in both. Savannas are, by definition, grasslands [29] and consist primarily of diverse grasses with scattered scrub. Forests, and the Mchelelo riverine forest in particular [27], have a diverse assortment of plant life, much of which is fruit and/or seed-bearing. Fruits and seeds are commonly recognized as being high in carbohydrates (i.e., energy) and lipids respectively while grasses are recognized as being high in protein and fiber [31]. If our predictions are supported, forest and savanna items should present as different clusters within right-angled mixture triangles (RMT), possibly representing either substitutable or complementary dietary choices [32]. We also predict a generally higher mineral content in the savanna food items–again because this group contains more grasses/legumes [33]–and the very limited available data comparing forest to savanna plants indicate savanna items may be richer in several of the minerals we assayed [34].

Finally, we predict that within the Mchelelo baboons’ top 15 food items (representing almost 50% of our food consumption observations collectively), we should see similar patterns as predicted above for all forest vs. savanna foods since they are a subset. However, some of those differences may be more extreme–either due to real distinctions or to the effects of a small N.

## Materials and methods

As described previously [19], the first author and assistants collected observational data (*N* = 55 months, 875 observation days, 4,893 hourly food consumption and troop location scans) [35] on the Mchelelo troop (*N*≈75 members) at the TRPNR (1°52'6"S, 40°8'3" E) between January 1988 and October 1992. The scans recorded genus/species of foods being consumed and broad category of the item (i.e., fruit/seed, corm/shoot, flower). A detailed study group description and map of the site can be found in [36–38]. The scan observations were used to produce a preliminary food inventory [19] that formed the basis of the list of foods for the nutrient analyses. The scan observations were distributed throughout the day and across months/seasons over the 55-month period and we believe they provide valid insight into the troop’s foods. All observational research complied with protocols approved by the Emory University, Atlanta, GA, IACUC. All aspects of this project conformed to Kenyan and US legal requirements and applicable national laws, and adhered to the American Society of Primatologists’ Principles for the Ethical Treatment of Primates.

At the same time, it should be recognized that feeding behaviors were not a focus of the baboon project between 1988 and 1992 and the feeding data are thus less detailed and specific than those reported by other projects where such information was the central focus. The feeding data are based entirely on the hourly scans. We collected neither fecal nor urine samples, conducted no time studies, calculated no consumption rates, and made no biomass estimates or accountings of either food availability or variation in use of plant species or parts. Thus, we are limited by the nature of the data in our abilities to characterize dietary diversity and selectivity for the Mchelelo baboons or to compare our data to other populations or species. A lack of intake data means we cannot address nutrient balancing. We also note that some spatiotemporal variation in nutrient content is likely given the 16–20 year gap between behavioral observations and plant sample collection. Nonetheless, our data provide an important and new record of many of this population’s foods and their respective nutrient compositions.

Our Kenyan field assistant, Hassan Jillo, who knew and could easily identify the plants, collected our samples from areas where consumption had been observed. These samples were collected between April 2008 and March 2009. None of the samples were from threatened or endangered plant species. The samples were air dried on a screen in a protected location out of direct sunlight at the TRPNR and stored in zip-lock bags until being shipped in September and December 2008 and March 2009 to the Nutrition Laboratory, Smithsonian National Zoological Park, Washington, DC, (NZP) for analyses.

All collections and shipments were conducted in accordance with Bentley-Condit’s research authorization from the Republic of Kenya (MOST-13/001/38C), Kenya Wildlife Services research permit (KWS/SC&M/5001) and KWS Export Permits (008040, 008156, 008158), and United States Department of Agriculture Import Permit (PDEP-08-00070).

Although not all items the baboons were observed to eat in 1988–1992 were available for collection in 2008–2009, we were able to obtain and analyze 118 samples representing 56 flora species and 31 families for our comparison of forest to savanna items. Where food items were observed to be consumed in both forest and savanna locations, we analyzed samples from both. We therefore have macronutrient data for 34 forest species (plus one from the available literature) and 24 savanna species (plus one as previous). Collectively, these represent 47 fruit/seed, 7 corm/shoot, 6 seed, and 2 flower/bud samples. For mineral content, we have slightly fewer samples– 29 species (plus one as above) from the forest and 20 species (plus two from the literature) from the savanna. The difference between macronutrient and mineral analyses was due to insufficient sample upon which to run the mineral assays for some species. The two “plus” items mentioned above were included because one represented one of the baboons’ top fifteen foods and the other had easily accessed mineral data to supplement our assayed macronutrient data.

Also included as a subset of our data are the Mchelelo troop’s top fifteen foods. The top 15 foods are examined independently because they, individually, represent all of the identified food items consumed by the Mchelelo troop that accounted for at least 1% of our observations and because we wanted to determine whether they, collectively, are different from the other food items. We chose 1% as it is the commonly accepted limit used in other studies (e.g., [8]). One additional corm/grass, *Brachiaria pubifolia* (also known as *Brachiaria xantholeuca*) was tied for 15^th^ place on our list. However, we had no sample of this particular item which we could analyze and we were unable to find nutrient content data that we could use. Thus, this sixteenth food item, which would only have added an additional 1.03% of observations to the dataset, is not included in our analyses. The 15 food items, collectively, accounted for 49.4% of our food consumption observations.

### Crude protein

All samples were processed and analyzed via chemical assays using AOAC [39] standard techniques for plants (see also [40]) and all results were calculated on a dry matter basis (%DM). Dry samples were first ground using a Wiley mill through a 1 mm screen. Nitrogen was measured following a Dumas nitrogen gas analysis procedure using a carbon, hydrogen, and nitrogen (CHN) elemental gas analyzer (Model 2400, Perkin Elmer, Norwalk, CT). CHN subsample vials containing 3-7mg of sample were dried at 100°C in a forced air convection oven for three hours. The ratio of dry weight to original weight was used as a dry-matter correction factor for the other assay results. CHN subsamples were run in triplicate and the nitrogen results were multiplied by the traditional factor of 6.25 to determine crude protein (i.e., % DM crude protein = %DM N x 6.25) [41]. However, we note that factors between 4.3 and 5.64 have been suggested as alternatives for tropical foliage [42–44] so 6.25 may lead to an over-estimation of crude protein. We also note there are limitations to using crude protein rather than available protein [45–46] but the NZP lab lacked the facilities for measuring available protein.

### Fiber

Neutral detergent fiber (NDF) and acid detergent fiber (ADF) assays were run non-sequentially on subsamples of approximately 0.12g following standard van Soest fiber procedures [47]. The non-sequential sampling was due to both small samples and the impracticality of extracting the NDF residue from a funnel crucible.

### Lipids

Lipid content was determined following Soxhlet fat extraction procedures where 1–1.5g of subsample duplicates were refluxed in 100 ml petroleum ether (boiling point 40° – 60°C) for 18 hours to extract the fat. Approximately 1.1g of each sample was ashed for four hours at 550°C to determine total mineral content (Thermolyne Furnatrol Series 133, Type 1700 Muffle Furnace, Sebron Corp., Dubuque, IA).

### Ash and minerals

Ash was solubilized in boiling nitric and perchloric acids and diluted with high-purity distilled water. Phosphorus content was assayed by the AOAC-Modified Gomorri colorimetric method with absorbance measured at 450 nm by an ultraviolet spectrophotometer [39, 48]. Calcium, iron, magnesium, manganese, potassium, and zinc were measured using flame atomic absorption spectroscopy (Perkin Elmer AAnalyst 800 Spectrophotometer, PerkinElmer Life Sciences, Shelton, CT). We specifically chose not to attempt to measure sodium as so much depends on the soil concentration which is highly variable even within a locale, it tends to concentrate locally, and it is affected by rainfall [49–50] and we had no way evaluate the Tana soils.

### Gross energy

Gross energy content (GE) on a dry matter basis was determined by drying approximately 0.5g plant material pellets at 100°C in a forced air convection oven for one hour and then combusting those pellets in a pure oxygen atmosphere in an adiabatic bomb calorimeter (Parr 1241 Adiabatic Oxygen Bomb Calorimeter, Parr Instrument Co., Moline, IL) with the heat produced measured in kcal/g. We recognize that GE exceeds the metabolizable energy (ME) that is actually available to the individual due to losses in both feces and urine. However, we agree with Hohmann et al. [51] that GE presents an important starting point for comparing the potential importance of different macronutrients in foods.

Further, the plant constituents that we measured (ash, NDF, fat and crude protein) did not comprise 100% of the dry matter of any of the plant foods assayed. The unmeasured portion will largely consist of carbohydrate molecules (including simple sugars, starch, pectins, fructans, β-glycans, and other oligosaccharides), although there will also be non-carbohydrate molecules such as tannins, other plant secondary compounds, and other molecules important to plant metabolism. The fraction calculated by the formula: 100 –(NDF + fat + CP + ash), has been variously termed nonstructural carbohydrates (NSC) [39] and total nonstructural carbohydrates (TNC). There are chemical assay methods to determine nonstructural carbohydrate content directly [52], but those assays were not possible at the NZP Nutrition Laboratory, and even these analytic methods have been shown to produce variable results depending on the assay and the laboratory [53]. Although the calculated estimates appear sufficient for pasture grasses, silages, and other feeds given to cattle and horses [47], these plant foods generally contain low levels of tannins and other plant secondary compounds.

We hypothesized that wild plant foods were likely to contain greater quantities of non-carbohydrate material (e.g. tannins) in the calculated TNC fraction. We tested this hypothesis by comparing the fraction of GE unexplained by the measured plant constituents, assuming values of 9.1 kcal/g for fat, 5.8 kcals/g for CP and 4 kcal/g for NDF [41] with the amount of TNC. If TNC was largely composed of carbohydrates, then the ratio of unexplained GE (Eq 1) to TNC (Eq 2) would be approximately 4 kcal/g.

GE−(GEfromfat+GEfromCP+GEfromNDF)=GEofTNC (1)

TNC=100−(fat+CP+NDF+Ash) (2)

Our results generally were greater than 4 kcal/g, sometimes substantially so. What this tells us is that TNC contains some high GE substances that likely are not carbohydrates and, thus, calculating metabolizable energy (ME) based upon calculated TNC will not be accurate. Thus, GE, while accurate, has issues with biological meaning while ME, with admittedly more biological meaning, will not be accurate due to all of the assumptions that have to be made about the carbohydrate fraction. Ultimately, we prioritized accuracy and thus did not attempt to calculate ME. While we do not present data regarding TNC or calculate ME, we do incorporate non-protein, non-fiber energy sources into our right-angled mixture triangles on the implicit axis, as described below, to facilitate visualization of the data.

### Right-angled mixture triangles and statistics

The right-angled mixture triangles (RMT) are a geometric approach to presenting food item composition [32, 54]. They are primarily a heuristic device for plotting and envisioning nutritional data where the fourth axis, in this case, is implicit and read off the *I*-axis as (*I* = 100%—(*X* + *Y + Z*)) [32, 54]. Thus, the RMT presents a four-component mixture with coordinates (*I*,*X*,*Y*,*Z*) in three dimensional space[32, 54]. We plotted Ash on the *X*-axis, CP on the *Y*-axis, and NDF on the *Z*-axis. The implicit axis (*I*) represents non-protein, non-fiber energy sources. All RMTs were plotted using Sigma Plot 13.

The results of the individual assays described above were pooled to produce the summary data discussed in this paper and contained in the tables herein. All statistical tests use the non-parametric Mann-Whitney U test as the data for some of our nutrients are not normally distributed, even when ArcSin transformed. Significance level was initially set at α ≤ 0.05 and then adjusted using a Bonferroni correction to account for multiple comparisons to α ≤ 0.004. Statistical analyses utilized IBM SPSS Statistics 24.

## Results

The macronutrient and mineral composition of the Mchelelo troop’s assayed food items are presented in the following Tables. Table 1 and Table 2 focus on the macronutrient content of forest and savanna foods respectively, while Table 3 and Table 4 present mineral content data, again for the forest and savanna samples, respectively. In each of these, food items that are part of the top 15 foods are presented in bold. Ten items are in bold in the forest tables and eight in the savanna tables as three food items are found in both locations. The macronutrients in Table 1 and Table 2 are presented as % DM, GE is kcal/g, and the minerals in Table 3 and Table 4 are mg/kg.

**Table 1:** Mchelelo baboon dietary macronutrient content (%DM)–forest samples (all).

Family	Species	Food type	GE (kcal/g)	NDF %	ADF %	Crude Protein %	Ash %	Lipids %
***Alangiaceae***	***Alangium salviifolium***	**F/S**	**4.39**	**35.56**	**16.32**	**10.34**	**4.08**	**0.81**
***Anacardiaceae***	***Sorindeia madagascariensis***	**F/S**	**4.41**	**36.82**	**28.12**	**7.08**	**2.67**	**1.52**
*Apocynaceae*	*Hunteria zeylanica*	F/S	4.53	27.96	13.41	7.80	4.26	4.56
*Apocynaceae*	*Rauvolfia mombasiana*	F/S	5.22	42.39	27.22	11.84	6.52	14.50
*Apocynaceae*	*Saba comorensis*	F/S	5.13	37.89	28.23	6.48	4.37	7.52
***Boraginaceae***	***Cordia sinensis***	**F/S**	**4.32**	**32.48**	**25.28**	**11.82**	**5.01**	**4.69**
*Caesalpiniaceae*	*Tamarindus indica*	F/S	4.9	58.51	38.68	19.24	4.82	0.47
*Cucurbitaceae*	*Momordica trifoliata*	F/S	5.72	66.22	35.40	15.01	2.51	12.41
***Ebenaceae***	***Diospyros mespiliformis***	**F/S**	**4.68**	**59.45**	**46.41**	**3.73**	**4.91**	**0.68**
*Euphorbiaceae*	*Antidesma venosum*	F/S	4.53	57.86	51.23	5.49	5.81	4.29
*Euphorbiaceae*	*Securinega virosa*	F/S	4.89	39.70	33.24	9.60	6.60	12.28
*Fabaceae*	*Cynometra lukei*	F/S	4.43	51.26	36.78	5.81	4.97	0.49
*Flacourtiaceae*	*Oncoba spinosa*	F/S	5.09	44.83	35.71	7.29	3.90	11.27
***Guttiferae***	***Garcinia livingstonei***	**F/S**	**4.69**	**25.72**	**20.76**	**5.62**	**2.90**	**9.99**
***Mimosaceae***	***Acacia robusta***	**S**	**4.66**	**48.31**	**41.09**	**13.17**	**6.45**	**1.39**
*Mimosaceae*	*Acacia rovumae*	S	4.43	59.79	39.73	7.26	4.63	0.71
*Mimosaceae*	*Albizia gummifera*	F/S	4.68	73.36	63.90	13.41	5.26	0.51
*Moraceae*	*Ficus natalensis*	F/S	4.64	43.35	35.69	6.98	8.69	6.62
*Moraceae*	*Ficus sycomorus*	F/S	4.68	51.61	41.46	9.36	7.26	4.56
***Palmae***	***Hyphaene compressa***	**S**	**4.67**	**76.38**	**55.33**	**5.34**	**1.68**	**6.47**
***Palmae***	***Phoenix reclinata***	**F/S**	**4.74**	**54.64**	**48.11**	**4.99**	**3.72**	**1.37**
***Poaceae*** ***#***	***Cenchrus ciliaris***	**C/S**	****	**72.20**	**38.50**	**8.70**	**13.90**	**3.34**
*Putranjivaceae*	*Drypetes natalensis*	F/S	4.40	58.41	40.51	10.15	5.88	3.18
*Rubiaceae*	*Polysphaeria multiflora*	F/S	4.88	59.49	45.79	4.71	3.40	1.02
*Salvadoraceae*	*Azima tetracantha*	F/S	4.49	53.77	43.96	9.03	7.84	8.24
*Sapindaceae*	*Chytranthus obliquinervis*	F/S	4.60	36.47	24.81	11.08	4.39	5.55
*Sapindaecae*	*Deinbollia borbonica*	F/S	5.19	37.38	30.68	11.77	5.10	17.50
***Sapindaceae***	***Lecaniodiscus fraxinifolius***	**F/S**	**4.63**	**37.59**	**31.04**	**9.46**	**3.06**	**2.32**
*Sapotaceae*	*Mimusops fruticosa*	F/S	4.79	44.86	25.93	3.89	2.94	4.01
*Sapotaceae*	*Pachystela brevipes*	F/S	4.69	33.72	19.78	6.79	2.73	7.89
*Sapotaceae*	*Pachystela msolo*	F/S	4.72	39.74	24.51	6.42	3.71	6.70
*Simaroubaceae*	*Harrisonia abyssinica*	F/S	4.52	30.75	27.09	7.55	9.69	9.90
*Tiliaceae*	*Grewia densa*	F/S	4.55	71.05	56.43	10.20	6.37	2.57
*Tiliaceae*	*Grewia stuhlmannii*	F/S	4.54	45.96	45.79	5.74	5.05	0.19
*Vitaceae*	*Cissus rotundifolia*	F/S	5.30	44.60	30.02	12.03	5.99	11.73
**MEANS** (& STANDARD DEVIATIONS)			**4.73** (0.30)	**48.29** (13.43)	**35.63** (11.53)	**8.72** (3.38)	**5.17** (2.32)	**5.46** (4.56)

**Key**: C/S = corms/shoots; Fl = flowers/buds; F = fruit; S = seed

**#**—no sample available from TRPNR; data compiled from tables in [55–58]. Items included in top 15 foods are in bold.

**Table 2:** Mchelelo baboon dietary macronutrient content (%DM)–savanna samples (all).

Family	Species	Food type	Ge (kcal/g)	Ndf %	Adf %	Crude protein %	Ash %	Lipids %
*Boraginaceae*	*Cordia monoica*	F/S	4.66	51.30	45.33	13.55	6.46	3.69
***Boraginaceae***	***Cordia sinensis***	**F/S**	**4.74**	**47.22**	**39.36**	**9.89**	**4.93**	**4.69**
*Burseraceae*	*Commiphora campestris*	F/S	4.80	51.60	47.99	5.46	7.54	10.63
*Capparaceae*	*Thylachium thomasii*	Fl	4.84	43.21	10.23	21.41	4.27	3.71
*Capparaceae*	*Thylachium thomasii*	S	4.61	34.44	18.20	18.41	7.09	6.23
*Combretaceae*	*Combretum constrictum*	F/S	4.86	47.02	42.33	14.20	4.06	11.74
*Combretaceae*	*Terminalia brevipes*	F/S	4.45	63.34	45.46	5.78	2.90	1.34
*Cucurbitaceae*	*Lagenaria sphaerica*	F/S	4.70	53.94	42.41	7.45	6.36	9.35
*Cyperaceae*	*Cyperus alpercuroides*	C/S	4.39	61.85	4.81	7.44	7.72	1.05
***Cyperaceae***	***Cyperus digitatus***	**C/S**	**4.30**	**63.37**	**28.96**	**19.46**	**12.26**	**1.41**
*Cyperaceae*	*Cyperus rotundus—corm*	C/S	4.08	54.05	12.00	4.53	3.18	0.77
*Cyperaceae*	*Cyperus rotundus—grass*	C/S	3.76	66.64	30.89	11.21	12.95	0.58
***Malvaceae***	***Abutilon figarianum***	**Fl**	**4.41**	**68.43**	**49.43**	**11.36**	**6.89**	**2.08**
*Malvaceae*	*Hibiscus mircranthus*	F/S	4.57	50.92	38.02	15.82	6.22	3.06
*Mimosaceae*	*Acacia zanzibarica*	S	4.17	16.16	15.88	8.07	3.70	0.27
***Poaceae*** ^***1***^	***Cenchrus ciliaris***	**C/S**	****	**72.20**	**38.50**	**8.70**	**13.90**	**3.34**
*Poaceae*	*Cynodon dactylon*	C/S	4.11	69.63	34.65	12.25	8.03	0.98
*Portulacaceae*	*Talinum portulacifolium*	F/S	4.15	52.22	42.84	5.51	5.21	1.02
***Salvadoraceae***	***Dobera glabra***	**F/S**	**3.72**	**28.09**	**7.75**	**26.10**	**16.68**	**0.19**
***Salvadoraceae***	***Salvadora persica***	**F/S**	**5.00**	**28.52**	**23.51**	**21.88**	**22.84**	**21.40**
***Sapindaceae***	***Lecaniodiscus fraxinifolius***	**F/S**	**4.55**	**27.45**	**21.56**	**8.16**	**3.25**	**2.32**
*Sapotaceae*	*Manilkara mochisia*	F/S	5.41	31.80	21.46	9.41	4.84	20.00
*Strychnaceae*	*Strychnos decussata*	F/S	4.76	49.58	15.55	8.34	2.51	2.14
***Tiliaceae***	***Grewia trichocarpa***	**F/S**	**4.43**	**66.96**	**51.98**	**13.74**	**5.89**	**1.63**
*Tiliaceae*	*Grewia villosa*	F/S	4.62	64.90	46.73	9.91	4.07	1.84
*Violaceae*	*Rinorea elliptica*	S	4.39	58.36	44.13	9.18	4.56	0.97
*Vitaceae*	*Cissus rotundifolia*	F/S	4.57	30.99	23.82	7.43	7.97	11.73
**MEANS** (& STANDARD DEVIATIONS)			**4.50** (0.36)	**50.16** (15.16)	**31.25** (14.18)	**11.65** (5.52)	**7.27** (4.63)	**4.75** (5.65)

**Key**: C/S = corms/shoots; Fl = flowers/buds; F = fruit; S = seed

^**1**^- no sample available from TRPNR; data compiled from tables in [55–58]. Items included in top 15 foods are in bold.

**Table 3:** Mchelelo baboon dietary mineral content (mg/kg [DM])—forest samples (all).

Family	Species	Calcium (Ca)	Iron (Fe)	Magnesium (Mg)	Manganese (Mn)	Phosphorus (P)	Potassium (K)	Zinc (Zn)
***Alangiaceae***	***Alangium salviifolium***	**1688.00**	**268.80**	**1191.00**	**16.60**	**2509.90**	**16126.40**	**21.90**
***Anacardiaceae***	***Sorindeia madagascariensis***	**2448.00**	**149.00**	**1408.00**	**17.80**	**1489.50**	**10652.70**	**11.70**
*Apocynaceae*	*Hunteria zeylanica*	1860.00	124.95	1600.50	18.55	1745.55	18131.25	11.40
*Apocynaceae*	*Rauvolfia mombasiana*	319.67	353.60	2634.33	39.90	2184.50	20965.87	20.67
*Apocynaceae*	*Saba comorensis*	2382.00	246.80	158.00	23.20	1100.50	17025.00	16.00
***Boraginaceae***	***Cordia sinensis***	**1626.00**	**64.10**	**1113.00**	**8.40**	**3093.80**	**18539.90**	**24.70**
*Caesalpiniaceae*	*Tamarindus indica*	6654.00	319.10	1676.00	18.60	1356.30	14619.00	11.50
*Cucurbitaceae*	*Momordica trifoliata*	394.00	207.70	3098.00	15.60	5332.10	4625.70	25.60
***Ebenaceae***	***Diospyros mespiliformis***	**3803.00**	**110.25**	**1143.00**	**3.30**	**1698.40**	**11349.60**	**5.55**
*Euphorbiaceae*	*Antidesma venosum*	6093.33	252.13	2495.33	39.17	2266.53	16009.40	19.23
*Fabaceae*	*Cynometra lukei*	7691.00	130.60	1798.00	34.90	1788.30	12014.80	12.70
*Flacourtiaceae*	*Oncoba spinosa*	1414.67	243.00	1390.33	11.27	2323.10	16772.43	11.73
***Guttiferae***	***Garcinia livingstonei***	**1822.66**	**174.53**	**1161.33**	**17.67**	**1141.37**	**11041.77**	**16.50**
***Mimosaceae***	***Acacia robusta***	**7065.00**	**79.85**	**2749.00**	**14.35**	**2227.80**	**14841.60**	**27.30**
*Mimosaceae*	*Albizia gummifera*	7027.00	116.90	3325.00	19.40	1100.80	14508.50	18.80
*Moraceae*	*Ficus natalensis*	4642.50	468.20	2829.50	23.90	2586.70	20559.05	22.10
*Moraceae*	*Ficus sycomorus*	11067.50	207.05	3079.50	24.80	2713.00	24890.30	28.85
***Palmae***	***Hyphaene compressa***	**416.50**	**99.35**	**1378.50**	**11.80**	**2684.55**	**5542.00**	**18.25**
***Palmae***	***Phoenix reclinata***	**1101.50**	**132.75**	**1338.75**	**18.63**	**1377.00**	**14772.45**	**13.25**
***Poaceae*** ^**2**^	***Cenchrus ciliaris***	**3033.33**	**34.10**	**1500.00**	**101.60**	**8033.33**	**3300.00**	**26.10**
*Putranjivaceae*	*Drypetes natalensis*	454.00	196.00	1965.00	17.90	2033.30	17308.90	27.90
*Rubiaceae*	*Polysphaeria multiflora*	2357.00	108.05	2277.00	16.75	1530.25	15207.10	9.55
*Salvadoraceae*	*Azima tetracantha*	16541.00	896.30	4514.00	19.30	1879.00	12538.00	22.10
*Sapindaceae*	*Chytranthus obliquinervis*	3254.75	234.90	2308.50	12.55	2736.38	14576.13	28.20
*Sapindaceae*	*Deinbollia borbonica*	5723.00	539.40	2296.60	23.80	3474.44	13968.62	22.46
***Sapindaceae***	***Lecaniodiscus fraxinifolius***	**1208.00**	**361.00**	**1025.00**	**10.50**	**2052.30**	**11449.70**	**15.90**
*Sapotaceae*	*Mimusops fruticosa*	2668.00	85.70	1103.00	9.35	846.30	9749.00	2.40
*Sapotaceae*	*Pachystela msolo*	2084.00	104.60	1668.00	13.20	1164.30	9336.10	15.90
*Simaroubaceae*	*Harrisonia abyssinica*	696.67	2197.57	3091.00	61.20	3538.67	13217.90	17.60
*Vitaceae*	*Cissus rotundifolia*	4095.00	199.10	1947.00	13.90	2809.80	22271.80	16.80
**MEANS** (& STANDARD DEVIATIONS)		**3721.04** (3524.42)	**290.18**(392.82)	**1975.41** (894.76)	**22.60** (18.44)	**2360.59** (1393.66)	**14197.03** (4861.63)	**18.09** (6.72)

KEY

^2^- no sample available from TRPNR; data compiled from tables in [58–60]. Items included in top 15 foods are in bold.

**Table 4:** Mchelelo baboon dietary mineral content (mg/kg [DM])–savanna samples (all).

Family	Species	Calcium (Ca)	Iron (Fe)	Magnesium (Mg)	Manganese (Mn)	Phosphorus (P)	Potassium (K)	Zinc (Zn)
*Boraginaceae*	*Cordia monoica*	3417.00	91.05	1456.50	13.75	2754.00	23129.05	20.25
***Boraginaceae***	***Cordia sinensis***	**2039.50**	**428.35**	**1348.00**	**10.80**	**2985.24**	**17334.35**	**25.75**
*Burseraceae*	*Commiphora campestris*	8699.50	66.40	2096.00	8.95	3430.55	18465.10	12.20
*Capparaceae*	*Thylachium thomasii* (flower)	1486.50	104.40	1182.50	14.45	1171.30	17745.70	17.40
*Capparaceae*	*Thylachium thomasii* (seed)	2490.00	95.27	3059.00	19.17	2094.97	28082.03	19.43
*Combretaceae*	*Combretum constrictum*	2602.00	199.45	1604.50	14.40	2853.25	16851.10	24.65
*Combretaceae*	*Terminalia brevipes*	3943.00	160.00	1198.00	29.80	1128.20	8126.00	6.10
*Cucurbitaceae*	*Lagenaria sphaerica*	4002.00	1237.20	2541.00	42.50	5279.90	23327.90	29.80
*Cyperaceae*	*Cyperus alpercuroides*	3436.00	169.50	1994.00	10.70	3038.90	12339.50	22.90
***Cyperaceae*** ^**3**^	***Cyperus digitatus***	**4178.50**	**55.18**	**1556.00**	**197.57**	**2400.00**	**3100.00**	**41.29**
*Cyperaceae*	*Cyperus rotundus—corm*	1239.00	311.20	1015.00	15.20	1681.00	11390.10	12.90
***Malvaceae***	***Abutilon figarianum***	**569.00**	**405.73**	**3274.33**	**33.23**	**4521.10**	**16014.67**	**65.43**
***Poaceae*** ^***4***^	***Cenchrus ciliaris***	**3033.33**	**34.10**	**1500.00**	**101.60**	**8033.33**	**3300.00**	**26.10**
*Poaceae*	*Cynodon dactylon*	5932.00	210.30	2244.00	53.30	2109.00	15080.50	17.00
***Salvadoraceae***	***Dobera glabra***	**1458.00**	**114.55**	**9543.00**	**4100.55**	**2026.15**	**17728.05**	**55.75**
***Salvadoraceae***	***Salvadora persica***	**968.00**	**1023.80**	**2843.00**	**44.00**	**3023.90**	**8500.60**	**21.00**
***Sapindaceae***	***Lecaniodiscus fraxinifolius***	**1892.50**	**518.25**	**1168.00**	**13.40**	**2413.40**	**11705.40**	**17.85**
*Sapotaceae*	*Manilkara mochisia*	2778.00	798.70	1534.50	26.70	1605.40	14311.70	17.20
*Strychnaceae*	*Strychnos decussata*	848.67	113.53	1014.67	59.87	824.70	11479.50	3.97
*Tiliaceae*	*Grewia trichocarpa*	8806.00	150.50	3901.00	19.90	2847.00	17811.20	15.60
*Tiliaceae*	*Grewia villosa*	5370.33	608.13	1500.33	27.13	1994.03	10638.10	14.53
*Violaceae*	*Rinorea elliptica*	1079.00	259.75	973.00	25.70	1783.20	15602.35	22.70
*Vitaceae*	*Cissus rotundifolia*	11622.0	238.20	3865.00	13.40	2512.60	12449.60	10.30
**MEANS** (& STANDARD DEVIATIONS)		**3560.43** (2796.85)	**321.46** (314.26)	**2278.75** (1779.22)	**212.87** (829.83)	**2717.88** (1511.82)	**14544.02** (5839.58)	**22.61** (14.11)

KEY

^3^- no sample available from TRPNR; data compiled from tables in [61–63]. Items included in top 15 foods are in bold.

^4^- no sample available from TRPNR; data compiled from tables in [58–60].

Forest foods had higher mean levels of GE, ADF, Lipids, Ca, and Fe than savanna foods (see Tables 1–4). Savanna foods had higher mean values of NDF, CP, Ash, Mg, Mn, K, P, and Zn than forest foods (see Tables 1–4). However, only the differences in CP was statistically significant (U = 1073.50, df = 117, p = 0.001).

If we focus on the top fifteen food items (see Tables 1–4) comparing forest (N = 10) to savanna (N = 8), there were four nutrients which were significantly different. Savanna foods had higher mean values of CP (U = 35.00, df = 33, p = 0.000), Ash (U = 36.00, df = 33, p = 0.001), Mg (U = 34.50, df = 29, p = 0.003), and Mn (U = 27.00, df = 29, p = 0.001). As well, the forest foods had a higher GE level (U = 42.00, df = 32, p = 0.003).

While we predicted the savanna food items would be higher in CP as indicated above, those differences do not reside just amongst the grasses as we had hypothesized. There are, in fact, 12 items with >10% CP in the savanna foods and five of those are near or over 20%. Only three of the 12 with >10% CP are grasses. Comparatively, while the forest items also had 12 with >10% CP, only one approached the 20% level (see Table 5). Thus, the difference in CP levels between sampled savanna and forest food items does not seem to be due to one or two outliers and appears to reflect a general difference in these two locations that goes beyond grasses. As well, eight of the top 15 food items appear in the listing of food items with >10% CP. Again, we acknowledge the potential issues with utilizing CP over available protein measurements; however, these data appear to indicate the importance of protein in this population’s dietary choices.

**Table 5:** Mchelelo baboon forest vs. savanna food items with >10% CP.

FOREST	% CP	SAVANNA	% CP
*Drypetes natalensis*	10.15	*Cyperus rotundus*	11.21
*Grewia densa*	10.20	***Abutilon figarianum***	11.36
***Alangium salviifolium***	10.34	*Cynodon dactylon*	12.25
*Chytranthus obliquinervis*	11.08	*Cordia monoica*	13.55
*Deinbollia borbonica*	11.77	***Grewia trichocarpa***	13.74
***Cordia sinensis***	11.82	*Combretum constrictum*	14.20
*Rauvolfia mombasiana*	11.84	*Hibiscus mircranthus*	15.82
*Cissus rotundifolia*	12.03	*Thylachium thomasii (seeds)*	18.41
***Acacia robusta***	13.17	***Cyprus digitatus***	19.46
*Albizia gummifera*	13.41	*Thylachium thomasii (flowers)*	21.41
*Momordica trifoliata*	15.01	***Salvadora persica***	21.88
*Tamarindus indica*	19.24	***Dobera glabra***	26.10

KEY: Food items from top 15 are in bold. Items arranged in ascending CP % order.

We also compared the top 15 food items to the remainder of the food samples (N = 41 species) ignoring location, primarily to examine whether differences are there regardless of where the items are found. There were no significant differences between these two data subsets in either their macronutrients or minerals when location was not taken into consideration.

**Fig 1:**
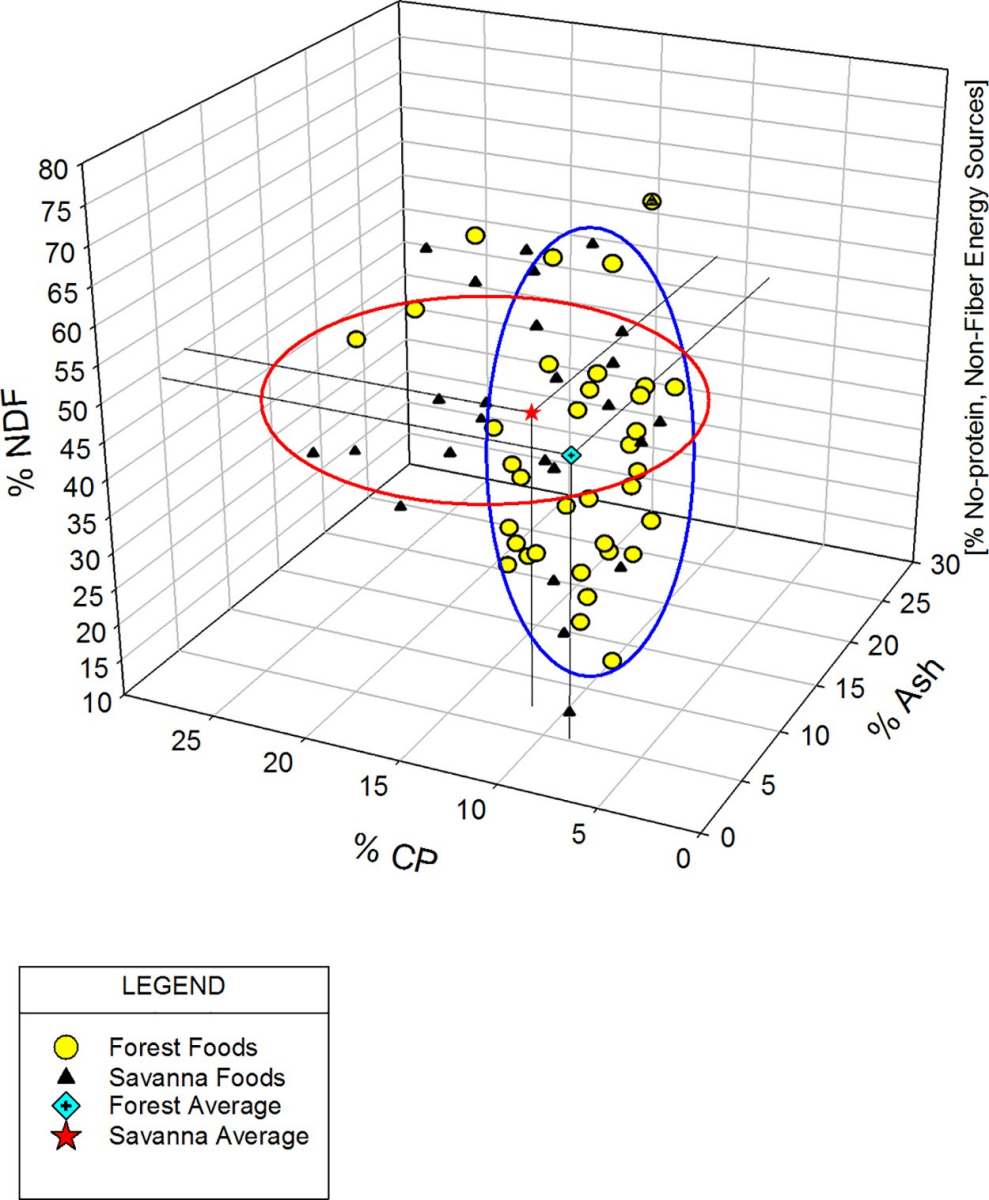
Mchelelo baboon forest vs. savanna foods macronutrient content–right-angled mixture triangle, all foods with cluster ellipses.

**Fig 2:**
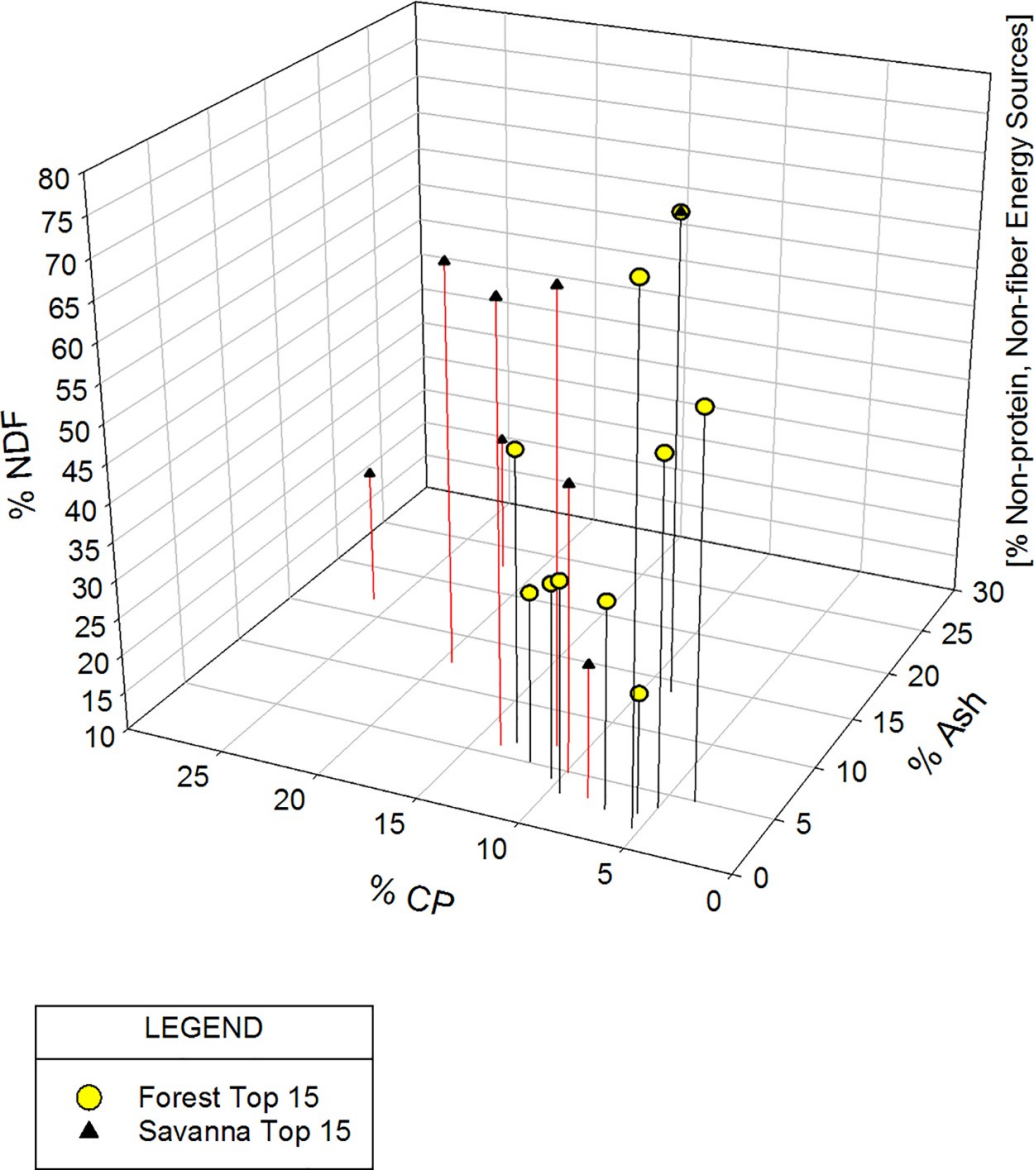
Mchelelo baboon forest vs. savanna foods macronutrient content–right-angled mixture triangle, top 15 foods.

To get a better sense of how the forest items compare to the savanna items in terms of their overall distribution of macronutrients, we plotted the data using a RMT [32, 54]. Each forest and savanna food was plotted independently in three-dimensional space in Fig 1. Fig 2 uses the same axes and procedures as Fig 1 to plot the top 15 foods, again with forest and savanna as indicated. The data-points in Fig 1 depict the foods based upon four broadly defined, primary components–minerals (as represented by ash–*X*-axis), fiber (as represented by NDF–*Z*-axis), protein (as represented by CP–*Y*-axis), and non-protein, non-fiber energy sources (on the implicit *I*-axis). There is substantial overlap between the two locations as can be seen in the distribution of the foods in three-dimensional space (also see Fig 1 ellipses overlap). However, there is also a difference. In both of these figures, but particularly in Fig 1, a clustering of the forest foods is apparent (see Fig 1 and Fig 2). Fig 1 illustrates both a greater clustering of the forest foods and a simultaneous greater spread of the savanna foods. This clustering is highlighted by the two identically-sized ellipses where we attempt to incorporate the most data-points. As can be seen, there are more savanna items outside of the savanna ellipsis than there are forest items outside of the forest ellipsis. Thus, the forest foods appear to be more similar to each other in their components than the savanna foods. The savanna items appear more variable.

Forest and savanna foods also differ in their overall averages and ratios. The averages are represented by the “diamond” and “star” symbols (forest and savanna respectively) with associated drop-lines. The average values for the forest foods are: ash = 5.17, CP = 8.72, NDF = 48.29, and non-protein, non-fiber energy sources = 37.82. The ratio for these values is thus 1: 1.7: 9.34: 7.3. The average values for the savanna foods are: ash = 7.27, CP = 11.65, NDF = 50.16, non-protein, non-fiber energy sources = 30.92 with a ratio of 1: 1.6: 6.9: 4.3. Not surprisingly given the differences reported in the previous paragraphs, savanna foods collectively have higher ash, CP, and NDF with lower carbohydrates while forest foods have lower values of ash, CP and NDF with higher mean carbohydrates. Thus, what this RMT (Fig 1) contributes is a visualization of these macronutrient data, again around four broadly defined components, that illustrates the pattern of the diversity between the two locations.

With the top fifteen food items plotted in Fig 2, we essentially see a repetition of the pattern in Fig 1. Because of the small dataset, we included drop lines to facilitate envisioning the pattern. Again, as represented in the statistically significant differences above, the RMT shows greater clustering of forest foods within the top 15 in their lower values of CP and ash with higher values expressed in the savanna foods. The higher ash values for the savanna foods likely reflect the differences found in Mn and Mg. As with the complete dataset, the RMT facilitates visualization of the differences between forest and savanna.

## Discussion

Tables 1 through 4 reveal that the Mchelelo baboons’ foods show a range of nutritional values for each of the macronutrients and minerals assayed. We predicted higher mean levels of GE and lipids in the forest foods and higher CP and fiber in the savanna items. While the mean values for the macronutrients varied in the directions we predicted, only the CP difference was statistically significant. When we limited our examination to the top 15 foods, our predictions were somewhat better supported. CP was significantly higher in those savanna foods in the top 15 as was ash (a general representation of mineral content) and the minerals Mg and Mn. As well, as predicted, GE was higher in the forest foods. Thus, while most of our nutrients did not meet our standard for statistical significance in the comparison of all foods, the differences were more pronounced when we focused on the top 15 as we anticipated might be the case. It is to these distinctions that we turn our attention.

Regarding the GE difference in the top 15, we indicated in the Methods section the difficulties in determining its biological relevance. We therefore simply note that the higher level found among forest foods is just a starting point for further exploration. However, we also note that this higher level of GE, and whatever proportion thereof represents ME, must necessarily influence the overall variation in the plotted distribution of forest and savanna foods in the RMTs discussed previously.

Determining the meaning of the mineral dissimilarities between the top 15 forest and savanna foods is complicated due to the complexity of micronutrient intake, uptake, interactions, and bioavailability, and is far beyond the scope of our study. While we predicted that the savanna items would be higher in mineral content than the forest and while those differences were represented in the mean values for five of the seven minerals assayed (see Tables 3 & 4), only Mg and Mn were statistically significant and only when we focused on the top 15 foods. As evidence of the complexity of utilization of these two minerals, we note others have shown animals deficient in Mg show higher uptake of Mn [64], Mn may be able to partially replace Mg in cell growth [65], Mn may displace Mg within cells [66], Mg may depress levels of Mn [67], and protein intake may positively influence Mg absorption [68].

The differentiation noted in the previous paragraphs, though limited, demonstrates the extent to which both forest and savanna areas are key to meeting the Mchelelo baboons’ nutritional needs. The difference in CP is particularly robust and, as presented previously, there are several food items that appear to contribute to this. Although we are unable to comment upon actual protein intake by the Mchelelo baboons, it appears that several items they consume have the potential to meet their protein requirements (adult primates: < 3g *BW_Kg_^-1^ *Day^-1^ or 6.4–8% of dietary dry matter [1]). While savanna items have a higher CP level than forest, the fact that there are several items in both locations with >10% CP (see Table 5) provides the Mchelelo baboons with options depending upon availability, for example, in the dry or wet seasons. It is thus plausible that, with these options, the Mchelelo baboons are able to maintain a relatively stable intake of protein across days/months/seasons as has been suggested for other monkeys [69]. These nutrient differences may tell us that the Mchelelo baboons go to the savanna to meet more of their protein and mineral needs and to the forests for more of their broad energy requirements. However, a more nuanced examination of both dietary intake and seasonal usage by the Mchelelo baboons awaits future research.

In addition to the forest-savanna divergences noted above, we find the overall similarity in forest and savanna foods (as demonstrated in the RMT overlap) intriguing. We infer that the overall similarities say something about the extent to which some of these items, or combinations of items, may be substitutable for one another by fulfilling similar needs. As mentioned previously, there is evidence for the importance of macronutrient balancing by various species [70–71]. Macronutrient balancing examines the ratios of protein, carbohydrates, and lipids within the diet and how animals may cope with shifts away from an ideal balance in varied and varying habitats [20, 70–71]. Our RMTs (see Figs 1 and 2) represent an examination of this issue for the Mchelelo baboons. Given the stated limitations of our data, our mapping of nutrients in these two figures is necessarily preliminary. However, the data show an overall distribution of macronutrient content–a general clustering of all the foods with a few outliers–that could plausibly represent macronutrient balance over time for this baboon troop. We note, in particular, the similar points in space represented by the forest and savanna “average” foods. We anticipate that future research at this site will be better able to address the issue of nutrient intake and macronutrient balancing by these baboons, how this group compares to others, and the interchangeability (or lack thereof) of particular food items.

Beyond its potential role in macronutrient balancing, Mchelelo baboon forest usage, in and of itself, is not particularly surprising. We expect baboons, like any other successful animal, to exhibit adaptations to their individual habitats. However, extensive forest usage has implications beyond baboon diet. First, there are the potentially negative implications for forest-dwelling species. There is, for example, substantial dietary overlap between the Mchelelo baboons and the endangered Tana River mangabeys (*Cercocebus galeritus*) within the forests [18, 72] and, thus, likely competition for these forest resources. Should further research find that the Mchelelo baboons utilize the forest for macronutrient balancing, that would present the opportunity for formulating predictions around nutrient availability and usage and the long-term implications for competition with other species such as the mangabeys (see Coogan and Raubenheimer [20]).

Second, there are potentially positive implications for the forest itself. The role of baboons as seed dispersers via intact seed in their feces has been noted at baboon research sites in two other countries (e.g., Ivory Coast [73]; Uganda [23, 74]). While we did not record the proportion of undamaged seeds in Mchelelo baboon feces, we did note seed presence. Overlapping with our research, Wieckzowski and Kinnaird [75] found an increase in tree abundance for six of seven tree species that were part of the Mchelelo baboons’ top 15 foods over a 13-year period in the Mchelelo West Forest. Although we cannot claim that the baboons were responsible for this increase, it is plausible that they may have played a role. Again, this aspect awaits further exploration.

Ultimately, we see our nutrient data as being the starting point for several lines of future research exploring such diverse topics as nutrient intake, maconutrient balancing, forest regeneration, and competition with other species over limited resources. We found both significant differences and interesting similarities between forest and savanna baboon food items. The next step is to determine what both the differences and the similarities mean for the baboons’ dietary choices and how those choices impact their habitat and other forest residents in this unique location. As we stated previously, there is still much to be learned about baboon nutrition.
